# Fungi rather than bacteria drive early mass loss from fungal necromass regardless of particle size

**DOI:** 10.1111/1758-2229.13280

**Published:** 2024-06-23

**Authors:** Eduardo Pérez‐Pazos, Katilyn V. Beidler, Achala Narayanan, Briana H. Beatty, François Maillard, Alexandra Bancos, Katherine A. Heckman, Peter G. Kennedy

**Affiliations:** ^1^ Ecology, Evolution, and Behavior Graduate Program University of Minnesota St. Paul Minnesota USA; ^2^ Department of Plant and Microbial Biology University of Minnesota St. Paul Minnesota USA; ^3^ Microbial Ecology Group, Department of Biology Lund University Lund Sweden; ^4^ USDA Forest Service Northern Research Station Houghton Michigan USA

## Abstract

Microbial necromass is increasingly recognized as an important fast‐cycling component of the long‐term carbon present in soils. To better understand how fungi and bacteria individually contribute to the decomposition of fungal necromass, three particle sizes (>500, 250–500, and <250 μm) of *Hyaloscypha bicolor* necromass were incubated in laboratory microcosms inoculated with individual strains of two fungi and two bacteria. Decomposition was assessed after 15 and 28 days via necromass loss, microbial respiration, and changes in necromass pH, water content, and chemistry. To examine how fungal–bacterial interactions impact microbial growth on necromass, single and paired cultures of bacteria and fungi were grown in microplates containing necromass‐infused media. Microbial growth was measured after 5 days through quantitative PCR. Regardless of particle size, necromass colonized by fungi had higher mass loss and respiration than both bacteria and uninoculated controls. Fungal colonization increased necromass pH, water content, and altered chemistry, while necromass colonized by bacteria remained mostly unaltered. Bacteria grew significantly more when co‐cultured with a fungus, while fungal growth was not significantly affected by bacteria. Collectively, our results suggest that fungi act as key early decomposers of fungal necromass and that bacteria may require the presence of fungi to actively participate in necromass decomposition.

## Graphical abstract

Dead fungal mycelium (also known as necromass) had higher mass loss, respiration, pH, and water content when colonized by fungi than by bacteria. When co‐cultured with a fungus, bacterial strains grew significantly more on necromass as the C source than when growing alone, while fungal growth was not significantly affected by bacteria.

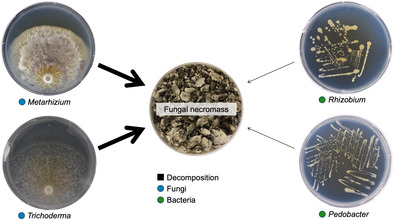

## INTRODUCTION

As fungal hyphae die and decompose, they create both “hot spots” (i.e., enhanced activity zones) and “hot moments” (i.e., enhanced activity times) in soil biogeochemical cycling (Brabcová et al., 2016; Kuzyakov & Blagodatskaya, 2015; McClain et al., 2003; Sokol et al., 2022). In forest ecosystems, more than half of soil organic carbon (C) and nitrogen (N) present is thought to be derived from fungal inputs (Angst et al., 2021; Clemmensen et al., 2013; Liang et al., 2019; Wang et al., 2021; Zhang et al., 2018), despite the fact that dead fungal mycelium (hereafter referred to as necromass) represents only a limited fraction of standing soil organic matter (SOM) stock (Dalal, 1998; Fogel & Hunt, 1983). This large contribution is due primarily to the rapid turnover of fungal necromass, with many field studies reporting greater than 50% mass loss occurring in the first month after senescence (Beidler et al., 2020; Brabcová et al., 2018; Fernandez & Kennedy, 2018; Ryan et al., 2020). Given this faster decay than other SOM pools such as plant litter (Beidler et al., 2021), it is important to understand early controls on microbial transformations of fungal necromass, as they likely have significant ecosystem‐scale consequences for biogeochemical cycling (Frey, 2019).

The microbial communities associated with decomposing fungal necromass are typically co‐dominated by taxonomically and functionally diverse groups of bacteria and fungi (Cantoran et al., 2023; Kennedy & Maillard, 2023). While sequence‐based microbial community profiling has been important in resolving the richness and composition of the fungal necrobiome (Beidler et al., 2020; Brabcová et al., 2016, 2018; Cantoran et al., 2023; Maillard et al., 2021, 2022), this method alone cannot directly detect which microorganisms are metabolically active at which times. Assessing the latter has been accomplished by isotopic labeling of fungal necromass, followed by tracking of isotopes into microbial cells (also known as stable isotope probing [SIP]). Laboratory‐based SIP studies comparing microbial incorporation of ^13^C‐labelled plant and microbial compounds have suggested bacteria are the dominant decomposers of fungal necromass, while fungi act as the dominant decomposers of plant litter (López‐Mondéjar et al., 2018, 2020). However, this classification is inconsistent with field‐based assessments of fungal necromass decomposition, where fungi and bacteria have been found to both uptake significant amounts of C and N from decaying fungal necromass (Maillard, Michaud, et al., 2023; Zeglin & Myrold, 2013). Resolving the relative contributions of bacteria and fungi in determining fungal necromass decomposition remains a key knowledge gap. As such, culture‐based studies offer a promising start for parsing the functional roles of fungal and bacterial decomposers and the conditions that may promote the dominance of one group over the other.

Prior to microbial transformations, physical fragmentation of organic matter by soil fauna facilitates the establishment of fungal and bacterial decomposer communities and initiates decay processes (Chapin et al., 2011; Prescott, 2010). According to surface‐area theory (Fricker et al., 2017; Glazier, 2005), the rate of any metabolic process (such as necromass decomposition) is limited by the rate at which resources can be transported from the surrounding environment into living cells. The multicellular nature of fungi provides them an advantage over unicellular bacteria during substrate colonization. Fungal mycelium exploit resources by means of hyphal extension while single‐cell bacteria are limited to smaller pore spaces in the soil (Ho et al., 2017; Yang & Van Elsas, 2018). For example, colony radial growth rates (*Kr*) for filamentous fungi are typically much higher than for bacteria (*Kr* (μm h^−1^) = 46–2152 in fungi, 18–29 in bacteria; Moore et al., 2020). Thus, bacterial ability to obtain resources from necromass is likely more limited and may rely more on diffusion than direct attachment in the absence of external physical or biological activities that augment their surface‐to‐area ratio. Under natural conditions, detritivore‐mediated fragmentation of organic matter increases the surface area for microbial colonization. In particular, nutrients in smaller organic matter fragments tend to leach faster than those in larger portions (Tiunov, 2009). Consequently, physical changes in the particle size and surface area of fungal necromass might impact the activity of both fungi and bacteria (Gupta & Germida, 2015; Seaton et al., 2020). Despite our rapidly growing understanding of how substrate chemistry affects rates of fungal necromass decay (See et al., 2021; Kennedy & Maillard, 2023), it is unclear how physical fragmentation of fungal hyphae and decomposer identity might interact to influence fungal necromass decomposition, especially at early stages of decay.

In this study, we evaluated the effect of necromass particle size (based on physical fragmentation) on fungal necromass decomposition, comparing two fungal and two bacterial strains from four genera that have been well documented to colonize decaying fungal necromass (Cantoran et al., 2023) and were selected based on differences in life‐history traits (Table 1). To include a range of hyphal fragments with increasing surface area‐to‐volume ratio, necromass was ground into coarse (>500 μm), intermediate (250–500 μm), and fine (<250 μm) particle sizes. Decomposition was assessed at each particle size category by measuring necromass loss after 15 and 28 days, thus spanning the most dynamic month of decomposition. In conjunction we evaluated different metrics of microbial activity, including respiration, growth, and changes in necromass properties (pH, water content, and chemistry). We hypothesized that (i) necromass decomposition by bacteria would increase with decreasing particle size (due to the increased surface‐to‐area ratio of smaller particle sizes), but that fungal decay rates would not be affected and (ii) total necromass remaining would not differ by particle size but would differ by both microbial type (fungi or bacteria) and strain after 28 days. To assess whether bacteria and fungi benefit from growing together, we performed a second experiment in which necromass was inoculated with single and paired cultures (i.e., bacterium and fungus), and relative growth (by means of their abundance in rRNA copy numbers) in each treatment was compared. We reasoned that fungi might modify the physical and chemical properties of necromass and thus promote bacterial growth. Therefore, we hypothesized that bacterial growth would be higher when co‐cultured with a fungal strain than when growing alone, but fungi would not have significant differences in growth when growing alone or in co‐culture with a bacterium.

**TABLE 1:** Species‐specific traits of the four microbial strains used in this study. Strains are shown in parenthesis next to the genus name and were originally isolated from *Hyaloscypha bicolor* necromass decomposed in a field setting. NCBI Accession corresponds to the highest similarity percentage sequence in GeneBank for ITS (fungi) and 16S (bacteria) regions. Lineage was assigned using BLAST results. To determine radial colony growth over time, 5 μl of initial cell slurries were pipetted onto the centre of 3% PDA plates. Plates were imaged each day and diameter growth plotted over time. Trophic mode was assigned from the literature (Karlsson et al., 2017; Leger & Wang, 2020; Woo et al., 2023). Images correspond to (left to right) *Trichoderma* (48WB1), *Metarhizium* (26WD1), *Rhizobium* (155), and *Pedobacter* (104) and were taken from 100 mm PDA and TSA plates for fungal and bacterial culture, respectively. *H. bicolor* mycelium had a C% of 46.64 (±0.23), N% of 4.51 (±0.04), and a C:N of 10.3 (±0.04). All C and N values are means of the percentages obtained from three independent replicates. Values in parenthesis represent ±1 standard deviation.

	*Trichoderma* (48WB1)	*Metarhizium* (26WD1)	*Rhizobium* (155)	*Pedobacter* (104)
Lineage	*hamatum* (sect. *Trichoderma*) Hypocreales	*marquandii* Hypocreales	*lusitanum*/*rhizogenes* Alphaproteobacteria	*nutrimenti* Sphingobacteriia
NCBI accession	MW269172	KT582066	MN712240	NR_133811
Growth speed	270 mm day^−1^	31 mm day^−1^	41 mm day^−1^	37 mm day^−1^
Trophic mode	Endophytism, Saprotrophism, Mycoparasistism	Endophytism, Saprotrophism, Mycoparasistism	Endophytism, Saprotrophism	Endophytism, Saprotrophism
Asexual spore type	Thin‐walled chlamydospores	Thick‐walled chlamydospores	Non‐spore forming	Non‐spore forming
% C	45.56 (±0.51)	44.62 (±0.3)	45 (±0.15)	42.48 (±0.73)
% N	4.53 (±0.04)	5.38 (±0.09)	4.06 (±0.08)	7.57 (±0.29)
C:N	10.1 (±0.04)	8.3 (±0.19)	10.5 (±0.19)	5.9 (±0.15)
	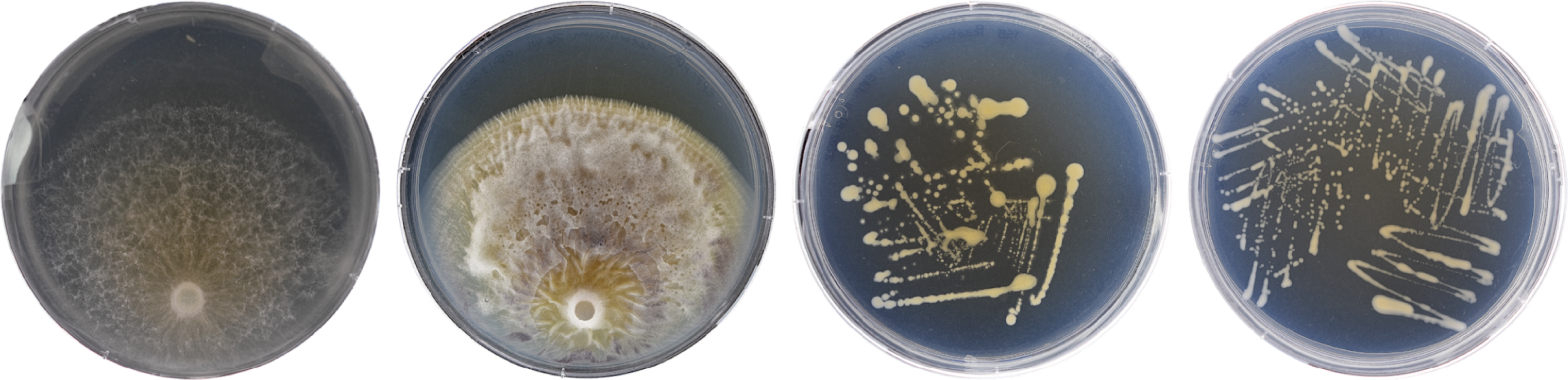			

## EXPERIMENTAL PROCEDURES

### Experiment 1

#### Strain selection and experimental design

To assess the effects of microbial identity on fungal necromass decomposition, we compared four microbial strains, two fungi and two bacteria, plus one uninoculated control. Strains were selected from a culture collection of bacteria and fungi isolated from decomposing fungal necromass incubated for 30 days in a *Pinus strobus*‐dominated forest in east central Minnesota, USA in Fall 2021. Strains differed in (i) growth rates, (ii) functional guilds, and (iii) growth morphologies (Table 1). The ITS1‐5.8S‐ITS2 region was amplified for fungal strains using ITS1F (Gardes & Bruns, 1993) and ITS4 (White et al., 1990) primers. For bacteria, the 16S region was amplified using 27F and 1492R primers (Weisburg et al., 1991). PCR products were Sanger sequenced in the forward direction with either ITS1F (fungi) or 27F (bacteria) primers at the University of Minnesota Genomics Centre. Sequences were then aligned to reference sequences using BLAST (Altschul et al., 1990), with the GenBank (NCBI) nucleotide collection database to assign identity based on high percentage of similarity. Since not all strains provided species‐level resolution (Table 1), we used the genus of each strain as the primary taxonomic designation. In referring to the microbes used in this experiment, we prefer to use strain when possible, considering (i) the high number of species present within each of these genera, and (ii) that other strains or species within these genera may lead to different results which were not tested in this study. To assess the effects of particle size on fungal necromass decomposition, three size ranges were compared: >500 μm, 250–500 μm, and <250 μm (see the procedures for the particle size in the *Necromass bag preparation* section). To determine the extent to which patterns of fungal necromass decomposition depended on incubation time, the experiment included two harvest times: 15 and 28 days. Thus, the full‐factorial design included (i) microbial strain (four levels), (ii) particle size (three levels), and (iii) harvest time (two levels), resulting in a total of 30 treatments (including the uninoculated controls). Five replicates were used per treatment, totaling 150 experimental units. Fifteen additional microcosms were designated to measure the effect of autoclaving on necromass leaching (five replicates per particle size).

#### Necromass production

*Hyaloscypha bicolor* (Hambl. & Sigler) Vohník, Fehrer & Réblová (formerly *Meliniomyces bicolor*) cultures were grown on half‐strength potato dextrose (PD; HiMedia Laboratories, PA, USA) agar (PDA) plates covered with a gel drying film (Promega, WI, USA) to avoid introducing agar plugs into liquid cultures. *H. bicolor* cultures were maintained in dark conditions, at 23°C, for 3 weeks. Next, mycelial plugs were transferred to liquid PD broth and the pH was adjusted to 5 using 10% hydrochloric acid (HCl). Cultures were grown in 125 ml Erlenmeyer glass flasks filled with 40 ml of liquid medium and incubated on orbital shakers at 150 rpm for 30 days at 25°C. After the incubation period, *H. bicolor* mycelium was harvested in sterile sieves and rinsed with sterile deionized water (diH_2_O) to remove traces of the liquid medium. Next, the mycelium was homogenized using a mortar and pestle, transferred to sterile 50 ml centrifuge tubes (Fisherbrand, PA, USA), and stored at −80°C overnight. Tubes were then placed into a benchtop freeze dryer (Labconco, NH, USA) for 3 days at −50°C under vacuum to create the necromass used in this study. Three independent necromass replicates were analysed for total C and N using an Elemental Analyser (Costech Analytical Technologies Inc), and for different compound classes by means of Fourier‐transform infrared spectroscopy (FTIR; see Fernandez et al., 2019 for full details regarding sample preparation).

#### Soil collection

Soil was collected from the same *Pinus strobus* forest from which the microbial strains were isolated at the Cedar Creek Ecosystem Science Reserve (MN, USA) in Summer 2022, to use as an incubation medium for decomposing necromass. Approximately the first 30 cm of soil was sampled with a surface‐sterilized shovel (ETOH 70%) after removing the litter layer and O‐horizon. Soil was then brought to the laboratory and maintained at 4°C until further processing. In the laboratory, the soil was sieved (within a week from collection) using a 500 μm (35 mesh) stainless steel sieve (Science First, FL, USA) and dried at 60°C for 48 h.

#### Necromass bag preparation

**FIGURE 1:**
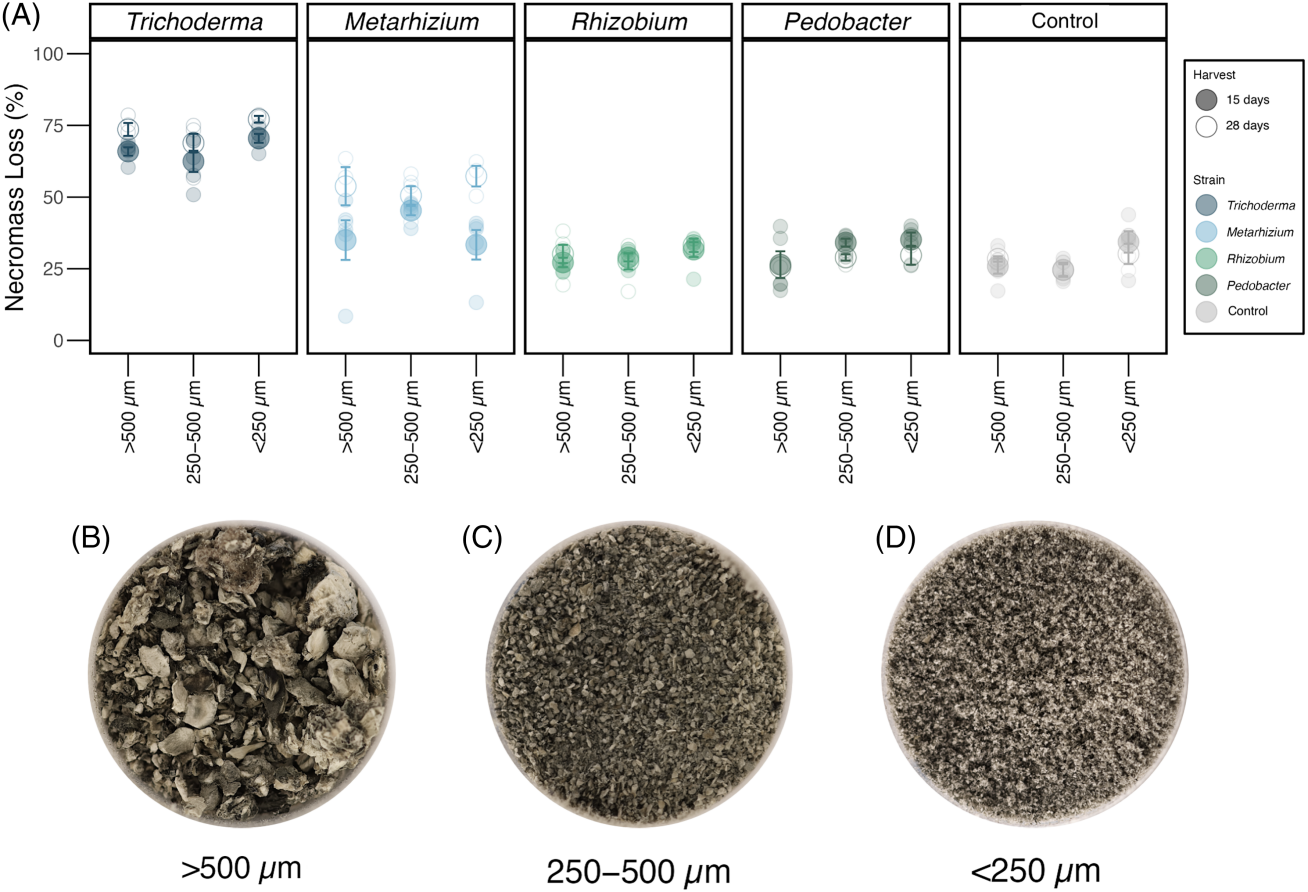
Fungal necromass loss (%) with different particle size by microbial strain. (A) necromass loss (%) at 14 days post‐inoculation (dpi) (solid circle) and 28 (hollow circle) dpi. Control panel shows the percentage of mass loss in uninoculated necromass. Error bars show ±1 standard error of the mean. Necromass particle sizes are shown for >500 μm (B), 250–500 μm (C), and <250 μm (D).

Freeze‐dried *H. bicolor* necromass was homogenized with a mortar and pestle and a subset was set aside to be used as the coarse particle size (Figure 1B). The remaining necromass was ground using a mortar and pestle and sieved to two particle size ranges: 250–500 μm (Figure 1C) and <250 μm (Figure 1D). Next, 52 μm polyester mesh bags (R510; ANKOM Technology, NY, USA) were filled up with 60 mg (±0.0004) of freeze‐dried necromass (hereafter referred to as mycobag) and heat sealed. A total of 60 mycobags per particle size were made.

#### Microbial inoculum preparation

Fungal cultures (*Metarhizium* and *Trichoderma*) were grown on PDA plates covered with a gel drying film for 3 weeks in dark conditions at 23°C. Fungal mycelium was then manually removed from the plate using a sterile 1000 μl pipette tip and transferred to 2 ml plastic tubes (Eppendorf, Hamburg, DE) containing 500 μl of diH_2_O. Next, the mycelium was macerated using sterile plastic pestles attached to an electric motor for 2–4 min to separate the hyphae into a colloidal solution. Then, 500 μl of diH_2_O was added to each tube and vortexed for 5 min. Tubes were then centrifuged at 12,000 rpm for 30 s and the supernatant was transferred to clean plastic tubes to remove large pieces of mycelium and have a homogeneous solution to use for microcosm inoculations. The slurries were then transferred to autoclaved test tubes (KAP‐UTS, Bellco, NJ, USA) containing 5 ml of diH_2_O. Fungal slurries were adjusted to an optical density (OD) of 0.28 (±0.02) at 600 nm using a spectrophotometer (SPECTRONIC 20D, Thermo Fisher Scientific, Waltham, MA, USA).

Bacterial cultures (*Pedobacter* and *Rhizobium*) were grown on 10% tryptic soy agar plates for 5 days at 23°C. Then, 50 ml sterile culture tubes were filled with 10 ml of 10% tryptic soy broth and inoculated with the bacterial strains. Cultures were grown for 2 days on orbital agitation at 150 rpm, and 25°C. After this period, the final OD was measured at 600 nm. Bacterial slurries were normalised to an OD of 0.28 using diH_2_O and the tubes were centrifuged at 15,000 rpm for 5 min. The supernatant was then discarded and bacterial cells were washed twice with diH_2_O to remove any residual medium. Finally, the supernatant was discarded and bacterial cells were resuspended to the same final volume (10 ml) using diH_2_O. Prior to inoculation of the microcosms, both fungal and bacterial slurries were diluted 1:10 using diH_2_O.

#### Microcosm assembly

Glass jars (Ball, GA, USA) of 236 ml capacity were filled with 80 g (±0.47) of dry soil. Next, 16.4 ml (±0.11) of diH_2_O was added to each jar to bring the soil to a water‐holding capacity (WHC) of 65%. All jars were then closed with loosened lids and tin foil. Lids were modified by drilling a 1 cm in diameter hole in the centre and covered with a 20 mm diameter filter paper sticker (0.3 μm pores; Zonon, CHN) to allow for gas exchange. All jars were autoclaved at 15 psi and 121°C for 50 min and left to cool for 24 h at 25°C. A single mycobag was buried into the centre of the jar using a sterile spatula, making sure that the necromass contained was at the bottom of the mycobag to ensure full contact with the soil (Figure S1). All jars were covered and autoclaved again prior to microbial inoculations. Necromass remaining after the autoclave was used as the total necromass at time zero considering any leaching due to the sterilization procedure. In a sterile hood, all jars were inoculated with 1 ml of microbial slurry and 1 ml of diH_2_O, pipetted onto the top of the mycobag. Afterward, jars were closed tight and covered with tin foil. Throughout the duration of the experiment, all jars were incubated in a closed cabinet at 23°C, 50% relative humidity, and in dark conditions. Soils were maintained at 65% WHC by adding sterile diH_2_O on a weekly basis.

#### Respiration measurements

Carbon dioxide (CO_2_) efflux was measured on days 1, 5, 8, 15, and 28 post‐inoculation. A subset of three jar replicates (randomly selected) per strain and particle size (*n* = 35) were designated for respiration measurements during the length of the experiment. Prior to measuring, paper filter stickers were replaced by 20 mm rubber septa (Metoot, CHN) for gas sampling under sterile conditions (Figure S1C). Jar headspaces were flushed with CO_2_‐free air (Air‐Tite Products Co., VA, USA) and incubated for 24 h. During sampling, microcosm headspace was mixed with a sterile needle, and 5 ml of sample was injected into an LI‐7000 infrared gas analyser (LI‐COR Inc., NE, USA) to determine headspace CO_2_ concentration. Once respiration measurements were concluded, rubber septa were replaced again by filter papers under sterile conditions.

#### Necromass collection

At each harvest time, microcosms were harvested destructively by opening the jar, collecting the mycobags, and opening the bag using clean tweezers in sterile conditions. Next, the fresh necromass remaining was transferred to sterile 2 ml plastic tubes using a clean spatula. Total fresh necromass weight was obtained using an analytical microbalance (OHAUS, NJ, USA). Approximately 10% of the total fresh necromass weight was transferred to a second 2 ml sterile plastic tube to be used for colony forming units (CFUs) counting and pH measurements. The remaining necromass was stored at −80°C overnight and then freeze‐dried for 3 days at −50°C, under vacuum. The final dry weight (DW) was then measured using an analytical microbalance. To account for the 10% of fresh necromass used for CFU and pH measurements procedures, we calculated the fresh weight:DW ratio (FW:DW) by subtracting the 10% necromass FW from the total necromass FW, and then dividing it by the un‐adjusted (90%) of necromass DW. To determine the total DW, the total FW was divided by the FW:DW and the percentage of mass loss at each harvest time was calculated.

#### CFUs and pH measurements

At each harvest time, 500 μl of diH_2_O was added to each of the tubes containing 10% of the total necromass FW. All samples from control treatments were additionally macerated with plastic pestles for 2 min and vortexed for 10 s. Then, 500 μl diH_2_O was added to all tubes, mixed, and centrifuged at 12,000 rpm for 30 s. One‐hundred μl of the supernatant from each sample was used to make serial dilutions (1 × 10^−3^ and 1 × 10^−4^ for fungal and bacterial slurries, respectively). Then, 100 μl of each dilution (three replicates per slurry) was spread onto PDA plates and CFUs were counted after 48 h. The pH of the remaining supernatant was then measured using a micro pH electrode (OHAUS, NJ, USA) plugged into a pH meter (GeneMate Avantor, PA, USA).

### Experiment 2

#### Strain selection and experimental design

To test the reciprocal effects of fungi and bacteria on one other's growth, we used a microplate bioassay. For this experiment, freeze‐dried *H. bicolor* necromass was ground using a mortar and pestle and sieved to a single particle size range (250–500 μm). For this experiment we decided to use a single particle size range as we were interested in evaluating microbial growth in co‐culture rather than necromass decomposition by particle size (as in Experiment 1). Bacterial cultures were prepared as described for experiment 1. Fungal cultures were grown on modified Norkan's C (MNC) agar plates and covered with a gel drying film as described in experiment 1. All bacterial and fungal isolates were transferred into a common liquid growth medium of 20% (v/v) 5X Minimal Salts (M9) (Sigma Aldrich, St. Louis, MO, USA), supplemented with >0.001% (w/v) of MnCl_2_, ZnCl_2_, CuCl_2_, CoCl_2_, Na_2_MoO_4_, CaCl_2_, MgSO_4_, and FeCl_3_ and adjusted to a pH of 5 using 10% HCl. Bacterial slurries were centrifuged at 15,000 rpm for 5 min. The supernatant was then discarded and bacterial cells were washed twice with diH_2_O to remove any residual medium. The pellet was resuspended into the supplemented M9 media, with the OD of each bacterial and fungal slurry adjusted to 0.10 (±0.02) at 600 nm using a microplate reader (Molecular Devices, San Jose, CA, USA) followed by a 10‐fold dilution to be used for the inoculation. In sterilized 96 well round‐bottom microtitre plates (Cell Treat, Pepperell, MA, USA), the modified M9 media was supplemented with 5 g/L of the powderized necromass. Every microbial strain was inoculated alone, and bacterial–fungal pairs were inoculated together, leading to eight unique combinations each consisting of four replicate wells. Each inoculation contained 200 μl of necromass‐containing media and 25 μl of each microbial isolate slurry. The treatments in which a microbial strain was inoculated alone were supplemented with 25 μl of the modified M9 media to reach an equal volume.

Experimental controls were included that were not incubated with any microbial isolate. The 96‐well microtitre plates were sealed with a thin layer of parafilm and incubated on an orbital shaker at 200 rpm at room temperature for 5 days. After incubation, the content of each well was transferred into a sterile 1.5 ml tube containing two sterile 3 mm steel beads, using wide‐bore 1 ml pipette tips. The wells were then rinsed with 250 μl of sterile diH_2_O to ensure all content was transferred to the tube. A bead mill (Fisher Scientific, Waltham, MA, USA) was used to manually disrupt the well contents, beating each tube for 30 s at a speed of 1.20 m/s. A 20 μl aliquot of the homogenized solution was taken for DNA extraction. The REDExtract‐N‐Amp Kit (Sigma Aldrich) was used and consisted of adding 20 μl of extraction solution, heating for 10 min at 65°C and 10 min at 95°C, and then adding 50 μl of 3% bovine serum albumin. After extraction, all samples were stored at −20°C ahead of molecular analyses.

#### Quantification of microbial abundance

The abundances of bacteria and fungi were measured using quantitative PCR (qPCR), in a StepOne Real‐time PCR machine (Thermo Fisher Scientific). Bacterial and fungal DNA were amplified with the 1401F/968R (bacterial specific 16S rRNA) and FR1/FF390 (fungal specific 18S rRNA) primer sets, respectively (Cébron et al., 2008; Chemidlin Prévost‐Bouré et al., 2011). The qPCR reactions were carried out with 1 μl of 1:10 diluted template DNA, standard bacterial or fungal linearized plasmids (10^9^–10^3^ gene copies/μl) or molecular grade water (negative control), in a 20 μl reaction volume using iQ SYBR Green Supermix (Bio‐Rad, Hercules, CA, USA). Amplification conditions consisted of 5 min at 95°C, followed by 40 cycles of 20 s at 95°C, 30 s at the primer‐specific annealing temperatures (56 and 50°C for 16S and 18S rRNA, respectively), and 60 s at 72°C. To assess primer specificity, a final melting curve analysis was also performed from 70 to 95°C, with a temperature increase of 0.3°C/s. Primers targeting the bacterial 16S region were used on all wells containing bacterial inoculum, the initial bacterial inoculum, and experimental controls to account for any contamination with bacteria. Similarly, primers targeting the fungal 18S region were used on all wells containing fungal inoculum, the initial fungal inoculum, and experimental controls to account for any contamination with fungi. Quantification for all samples was based on averages of technical duplicates and expressed as numbers of bacterial 16S or fungal 18S copies present in each incubation. The data ranged from 10^9^ to 10^3^ gene copies/μl for each sample. These numbers were transformed to reflect the gene abundances present in the liquid media at the end of the inoculation. Specifically, the data were multiplied by a factor of 10 to account for the dilution done for qPCR, multiplied by a factor of 4.5 to account for the addition of 70 μl of reagents to 20 μl of DNA sample during DNA extraction, and multiplied by a factor of 2 to account for the doubling in volume that occurred when the wells were rinsed. This resulted in data ranging from 10^11^ to 10^5^ copies used in the statistical analyses.

### Statistical analyses

All statistical analyses and data visualization were performed in R (R Core Team 2022) and considered significant at *p* < 0.05. Data assumptions of normality were tested by examination of q–q plots on the residuals for all analyses. To account for a slightly imbalanced final jar count due to elimination of contaminated jars, type III analyses of variance (ANOVAs) were conducted using the “car” package (Fox & Weisberg, 2019) to compare the percentage of necromass loss among (i) microbial strain, (ii) particle size, (iii) harvest time, and their interactions. Post hoc Tukey HSD tests were conducted using the “emmeans” package (Lenth et al., 2020) to assess differences among treatment means. Because there was no significant effect of particle size on mass loss by microbial strain (*F* = 2.32; *p* = 0.103; Figure 1; Table S1), the data were pooled across particle sizes by microbial identity and harvest time to estimate necromass decay rate and respiration, as well as pH and gravimetric water content (GWC). To determine total cumulative CO_2_‐C loss (mg C respired) for each time point (days 1, 5, 8, 15, and 28), point measurements of CO_2_ efflux were integrated over time using the trapezoidal rule, which estimates the area under the curve of respiration rates over time by dividing the total area by sampling time interval into trapezoids, calculating their area, and summing them. We used a log transformation to normalize the cumulative respiration data. CFU counts were log‐transformed (log_10_) and compared by means of one‐way ANOVA. Necromass GWC was estimated by subtracting the total necromass FW from the total necromass DW and dividing it by the total necromass DW. Then, GWC means were compared using one‐way ANOVA. Deltas were calculated for mass loss, pH, and GWC by subtracting the value obtained in the control from the value obtained per variable and then dividing it by the value obtained per variable (the raw values per strain are shown in Figures S2–S5). Then, one‐sample *t* tests were used to test if the necromass mean delta in mass loss, pH, and GWC per strain differed from zero. To assess the growth of fungi and bacteria alone or in co‐cultures, we used one‐sample *t* tests comparing the mean delta of copy numbers of each microbe in co‐culture against the mean copy number of its single culture. Tests and confidence intervals were then calculated using the “tidyverse” (Wickham et al., 2019) and “magrittr” (Milton & Wickham, 2014) packages. Finally, the observed FTIR peaks were assigned to functional chemical groups based on prior studies (Fernandez et al., 2019; Maillard, Pflender, et al., 2023; See et al., 2021) and the abundance of each functional group was compared by one‐way ANOVA and one‐sample *t* tests against the control. Tables summarizing the results of each of the aforementioned ANOVAs and *t* tests are presented in Tables S1–S13.

## RESULTS

Initial amounts of leaching of mass (due to autoclaving) from necromass were greatest for the smallest particle size (*F* = 5.35; *p* < 0.05); however, particle size had no significant effect on fungal necromass mass loss at either harvest time (*F* = 2.32; *p* > 0.05; Figure 1, Table S1). Though mass loss did differ significantly by microbial strain (*F* = 72.06; *p* < 0.01; Table S2), there was also a significant interaction between microbial strain and harvest time (*F* = 6.3; *p* < 0.01, Table S2). When necromass was colonized with *Trichoderma* or *Metarhizium* (fungi), higher mass loss than the uninoculated control treatment was observed at both harvest times (Figures 1 and 3A), reaching 75% mass loss after 28 days for *Trichoderma* and 50% for *Metarhizium*. In contrast, when necromass was colonized with *Rhizobium* and *Pedobacter* (bacteria), mass loss values were not significantly different from each other or the uninoculated control treatment at either harvest time (Figures 1 and 3A).

**FIGURE 2:**
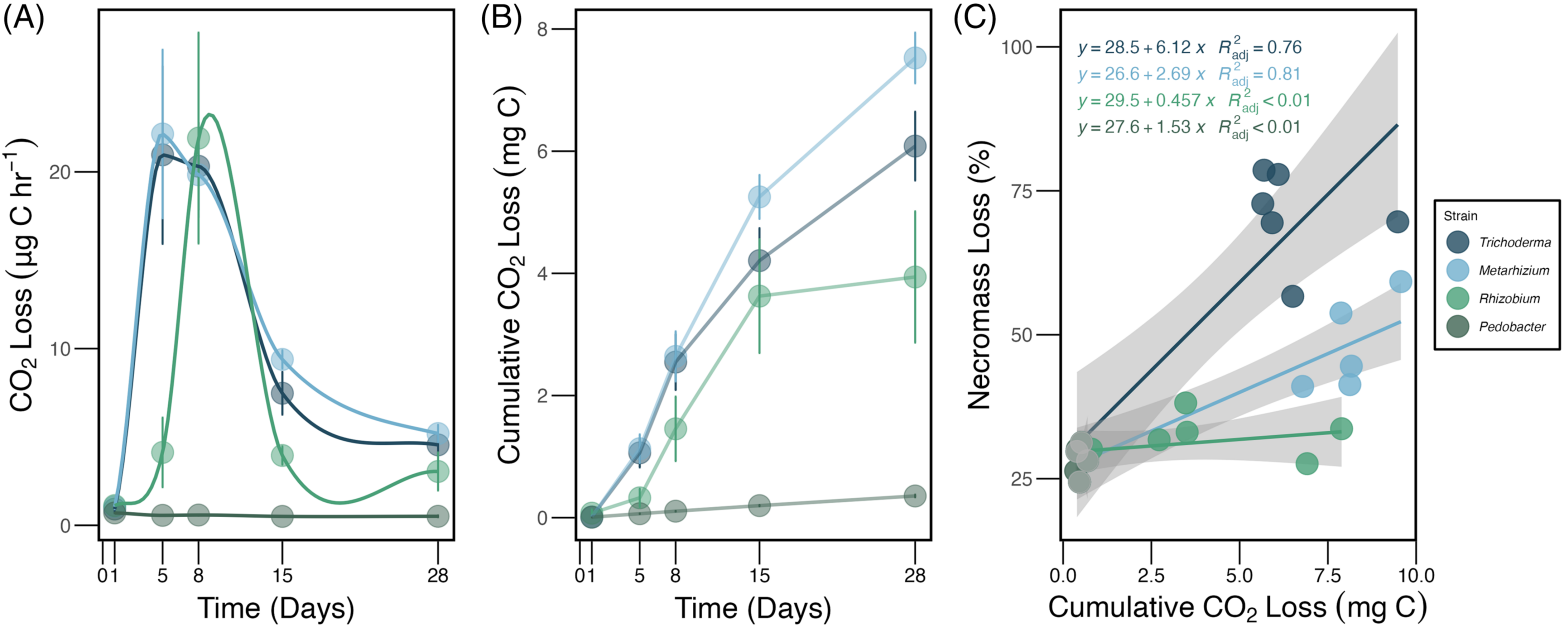
Respiration by microbial strain. (A) CO_2_ loss (μg C h^−1^) over time measured at 1, 5, 8, 15‐, and 28‐days post‐inoculation. (B) Cumulative CO_2_ loss by microbial strain. (C) Correlation between necromass loss (percentage) and cumulative CO_2_ (mg C) after 28 days by microbial strain. Lines in C shows the linear relationship (y = mx + b) between the variables for each microbial strain. At each time point, headspace CO_2_ concentration measurements were made after a 24‐h incubation period. Error bars show ±1 standard error of the mean (circles).

When necromass was colonized by fungi, the highest respiration was observed after 5 days (Figure 2A). Overall, cumulative respiration over 28 days was significantly higher for necromass colonized by fungi than that colonized with bacteria (Figure 2B, Table S3). Interestingly, while not contributing to mass loss, respiration values indicated that *Rhizobium* colonies were active during the length of the experiment, reaching the peak activity after 8 days of incubation (Figure 2A) and having a significantly higher cumulative respiration than that colonized by *Pedobacter* (Figure 2B, Table S3). Conversely, respiration measurements in *Pedobacter* treatments were not significantly different from uninoculated controls at any point, suggesting that this bacterium was not active during the incubation period. *Pedobacter* did, however, form colonies at the end of the experiment (Figure S4), excluding the possibility that the inoculum was non‐viable. Furthermore, when necromass was decomposed by fungi, there was a significant positive relationship between mass loss and cumulative CO_2_ production for both *Trichoderma* (*R* ^2^ = 0.76; *p* < 0.05) and *Metarhizium* (*R* ^2^ = 0.81; *p* < 0.05; Figure 2C). The same pattern was not present for necromass colonized with *Rhizobium* (*R* ^2^ < 0.01; *p* = 0.34) or *Pedobacter* (*R* ^2^ < 0.01; *p* = 0.89; Figure 2C).

**FIGURE 3:**
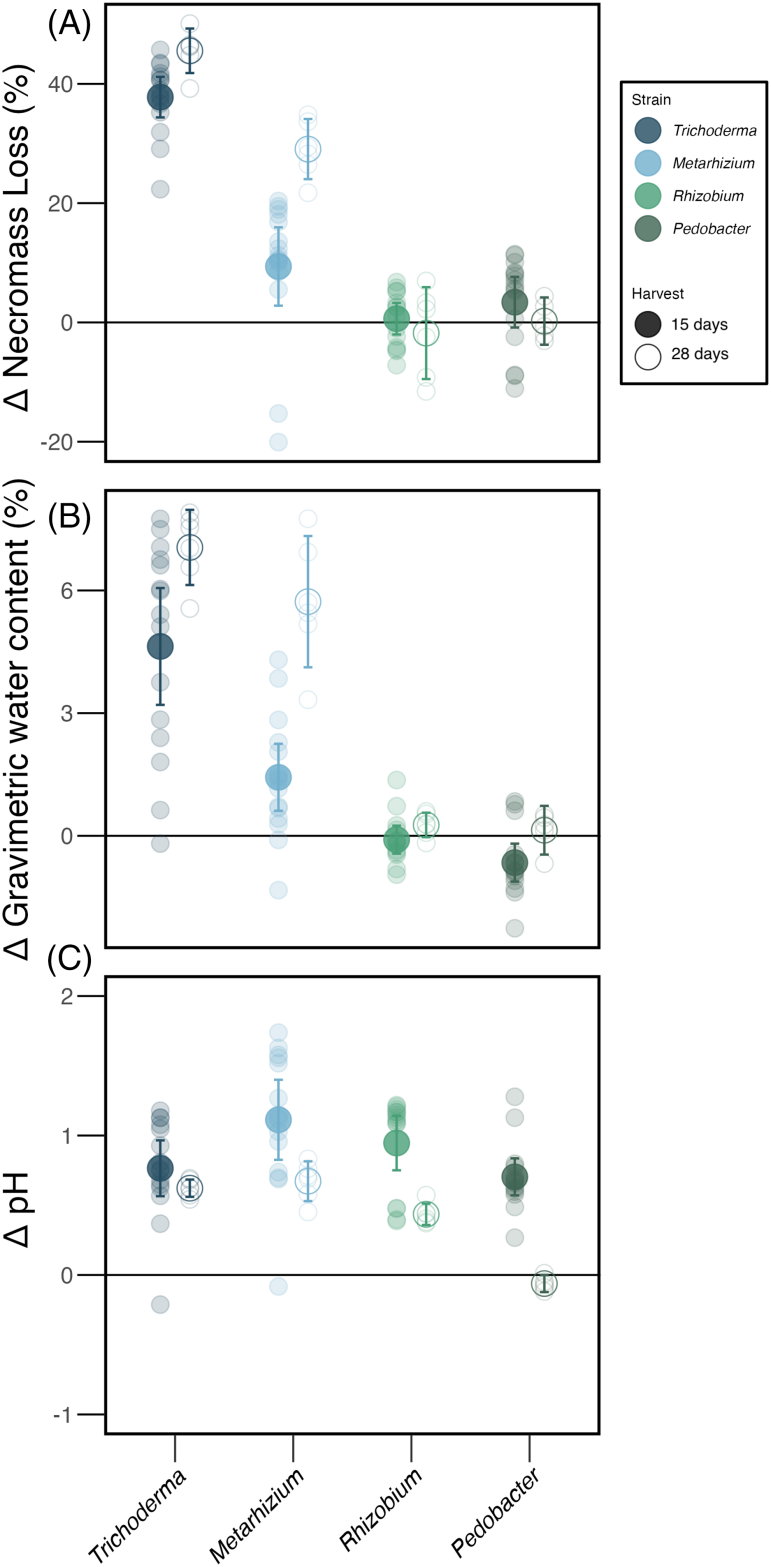
Microbial strain‐induced changes in necromass (expressed as delta “Δ”). Panel (A) necromass mass loss (%), (B) gravimetric water content (GWC) (%), and (C) pH. Values are shown by microbial strain at 15 days post‐inoculation (dpi) (solid circle) and 28 (hollow circle) dpi. Values are compared against the uninoculated control (0 line) at each time point (15 and 28 days). Error bars represent 95% confidence intervals.

GWC was significantly higher when necromass was colonized by fungi than by bacteria and there were also significant differences among strains (*F* = 37.29; *p* < 0.01, Figure 3B). These differences persisted after 28 days of incubation and were consistently higher for necromass colonized by *Trichoderma* relative to *Metarhizium* and both bacteria (Figures 3B and S3, Tables S4 and S8). There were also significant differences in pH by harvest day (*F* = 24.23; *p* < 0.01, Figure 3C) and strain (*F* = 27.46; *p* < 0.01, Figure 3C). The mean pH range for all taxa was 5.4–6.1, while the uninoculated treatments were at 4.9 after 28 days of incubation. Notably, the higher pH values were observed for necromass colonized by *Metarhizium* after 15 days (Figure S2, Tables S5 and S9). Finally, on average CFUs differed by strain (*F =* 23.70; *p <* 0.01) and *Rhizobium* CFU counts were higher than those for the other three microbial strains (Figure S4, Table S6).

**FIGURE 4:**
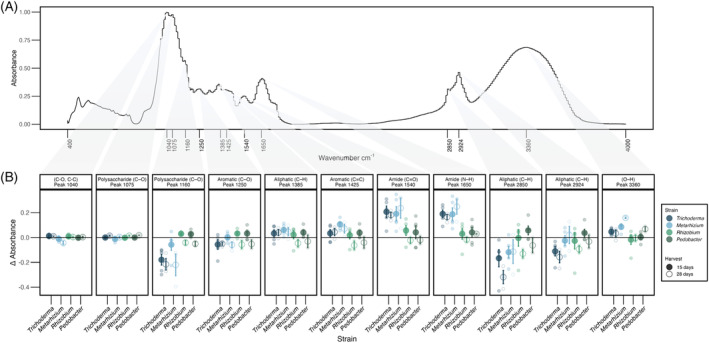
Absorbance values from Fourier‐transform infrared spectroscopy (FTIR) analyses on necromass colonized by each microbial strain. Panel (A) FTIR spectra showing the position of each peak; (B) absorbance values per peak showing peak position, the associated functional group (when available), and the chemical bond type on the faceted strip. Values are shown by strain at 15 days post‐inoculation (dpi) (solid circle) and 28 (hollow circle) dpi for the 11 peaks identified. Values are compared against the uninoculated control (0 line) at each time point (15 and 28 days). Error bars represent 95% confidence intervals.

We identified 11 peaks across all the FTIR spectra and significant chemical alterations of decomposing necromass were observed in five of them (Figures 4 and S5, Tables S11 and S12). In particular, when necromass was colonized by *Metarhizium*, a significantly higher abundance of amides (peaks 1540 and 1650) was observed after 28 days (Tables S11 and S12). Interestingly, *Trichoderma*‐colonized necromass was significantly depleted in polysaccharides (peak 1160) and aliphatics (peaks 2850 and 2924) when compared with *Metarhizium* (Tables S11 and S12). When contrasted against the control, *Metarhizium* and *Trichoderma* showed significant alterations in five and eight peaks, respectively, including amides, aromatic, and aliphatic functional groups (Figure 4, Tables S11 and S12). Both *Rhizobium* and *Pedobacter* had basically no effect across all peaks (Tables S11 and S12).

**FIGURE 5:**
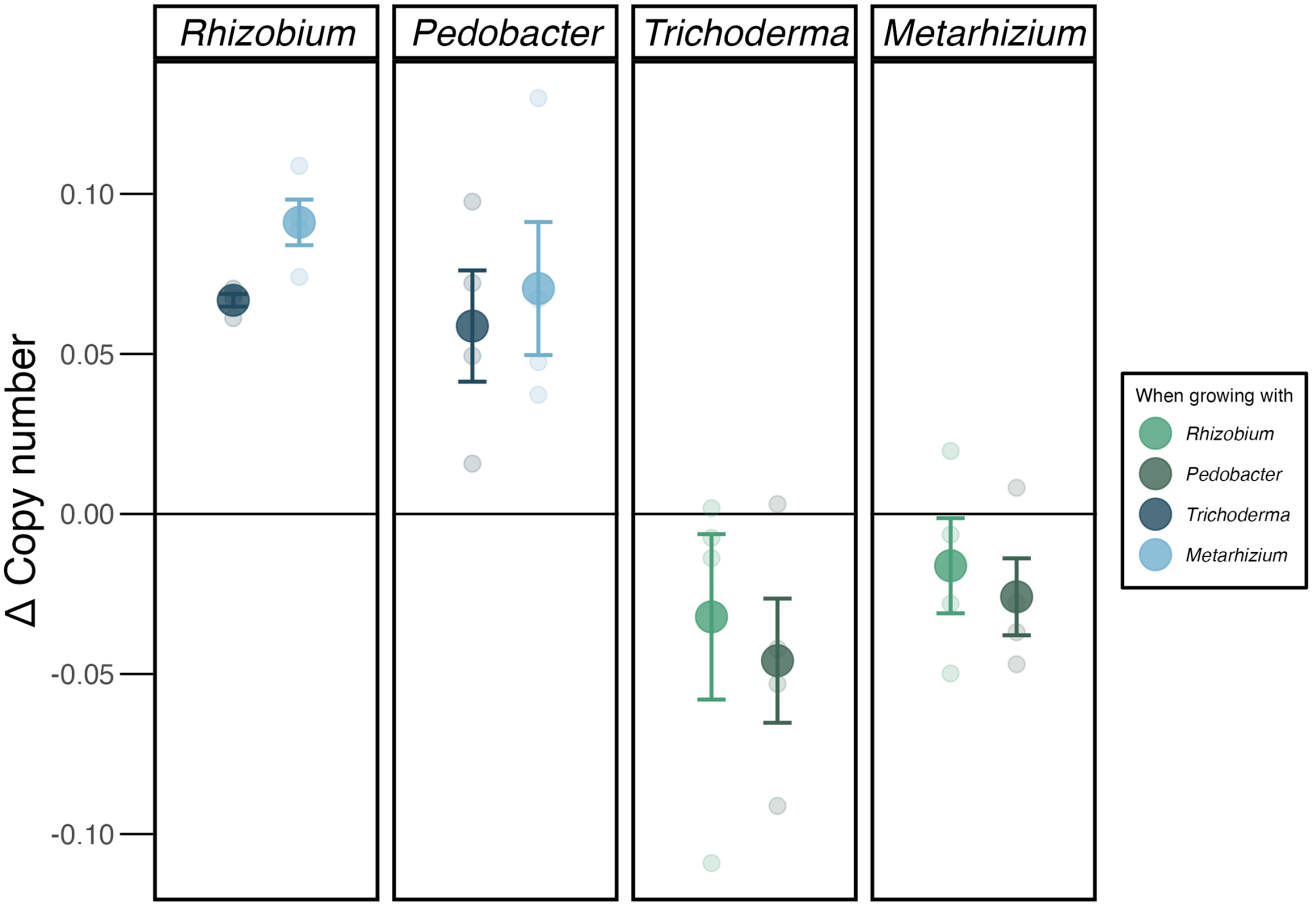
Microbial strain rRNA copy numbers in co‐culture (expressed as delta “Δ,” relative to when growing alone). Each panel shows the growth of each microbial strain (name on the strip of each panel) when growing with another strain (colours shown in the figure legend). Values, based on qPCR, were generated after a 5‐day incubation period. Error bars show the standard error. Values are compared against their values grown in isolation (0 line) at 5 days post‐inoculations. Error bars represent 95% confidence intervals.

Co‐inoculation with fungi benefitted both bacterial strains, resulting in higher bacterial abundance relative to growth alone (Figure 5). This result was demonstrated for *Pedobacter* grown with *Metarhizium* (*t* = 3.39, *p* < 0.05) and *Trichoderma* (*t* = 3.38, *p* < 0.05). Similarly, this result was significant for *Rhizobium* grown with *Metarhizium* (*t* = 12.78, *p* < 0.01) and *Trichoderma* (*t* = 33.76, *p* < 0.001). Though fungi appeared to grow less in co‐culture with bacteria, their growth did not differ significantly than when grown alone in all combinations (Table S13).

## DISCUSSION

In light of growing interest in fungal necromass decomposition as a significant contributor to soil C dynamics (Angst et al., 2021; Wang et al., 2021), this study builds on previous work done in mixed microbial communities by isolating the effects of individual microbial strains. While we did not find an effect of necromass particle size, we found that the early decomposition of necromass by fungi was significantly greater than by bacteria under standardized conditions, despite both microbial groups being found in high abundance on decaying necromass in a variety of ecosystems (Kennedy & Maillard, 2023). Additionally, we demonstrated that the enhanced mass loss caused by fungi was associated with a number of changes in the necromass substrate, including alterations in pH, water content, and residual chemistry.

The absence of an effect of particle size on fungal necromass decomposition rates was somewhat surprising, given the clear theory about the effects of surface area on resource access (Fricker et al., 2017; Glazier, 2005). Yet, our results are similar to other decomposition experiments testing the effects of substrate fragmentation on mass loss rates. Vestergaard et al. (2001), for example, found that finely ground (<2 mm) leaf litter and more intact pieces of both corn and barley leaves had similar decomposition rates and microbial biomass, suggesting the decomposer community abundance is not closely linked with physical fragmentation. Tiunov (2009), on the other hand, found greater mass loss and respiration from larger than smaller leaf particle sizes, which is the opposite of what would be expected about surface area and resource access. Further, Rinkes et al. (2014) found that ground leaf litter initially had greater microbial respiration than litter of larger fragment sizes, but that pattern disappeared after 86 h of incubation and also varied in magnitude depending on both litter species and soil type. Here, we observed that particle size of necromass did not have significant effects on mass loss or microbial respiration over 28 days, suggesting that the quality of the substrate overshadowed the effect of particle size. Collectively, these studies suggest that physical fragmentation of organic matter inputs, at least at the smaller scales investigated here (micrometre to cm), is not strongly predictive of decomposition rates. Our results are, however, important, as they clarify that the differences across studies of fungal necromass decomposition to date are robust for differences in particle size. Moreover, our results suggest that different fragmentation processes or variations in fungal structures (i.e., hyphae, mycelium, or sporocarps) are unlikely to lead to major consequences during the early stages of necromass decomposition in terrestrial ecosystems.

The differences in fungal necromass loss and chemistry by microbial type, while limited to only two fungal and two bacterial strains, were striking. Relative to the uninoculated control treatment, both fungal strains caused significant amounts of necromass loss at both harvest times, while neither of the bacterial strains did at either harvest time. The confirmation of viable microbial cells present on the necromass for all four strains at both harvest times (Figure S4) suggests that (i) our inoculum and inoculation methods were sufficient to establish new microbial populations and (ii) that bacteria and fungi were able to access the necromass in the mycobags (rather than being limited to the soil compartment only). Further, the presence of (i) measurable respiration and (ii) higher CFU counts in necromass versus sand (Figure S6) from at least one of the bacterial strains suggests that bacteria were not completely dormant after being added to the microcosms. Instead, our results are more consistent with a limited ability of bacteria to grow on necromass when isolated from fungi. Importantly, other studies have found that other bacterial strains can grow on fungal necromass (*Agaricus bisporus* and *Tylopilus felleus*) without fungi being present (Starke et al., 2020). That suggests that bacteria are capable of some degradation of fungal necromass independently, although the amount of degradation likely depends on multiple factors, including bacterial lineage, necromass chemistry, and the physical structure of the environment.

We suggest three features of fungal growth and physiology that likely contribute to their higher efficiency in accessing resources from fungal necromass than bacteria, especially early in decomposition. First, unicellular bacteria are largely confined to individual pore spaces in soil, while most multicellular fungi grow and branch in a filamentous fashion, exploiting resources across pore spaces through hyphal extension (Ho et al., 2017; Moore et al., 2020; Yang & Van Elsas, 2018). This difference may limit the opportunity for bacteria to encounter necromass, which is likely a patchily distributed resource within the soil matrix (Jensen et al., 2017). The faster respiration peaks observed for both of the fungal strains than *Rhizobium* in this experiment are consistent with more rapid fungal colonization of fungal necromass (Figure 2). Second, due to their hyphal growth form, fungi can penetrate into the areas they are decomposing, unlike bacteria, which are confined to outer necromass surfaces to which they simply attach (Lehmann et al., 2019; Lehmann & Rillig, 2015). This means that fungi should be able to more fully exploit resources present in patches of fungal necromass when encountered relative to bacteria that can only access the outer necromass surfaces. Finally, fungi produce a wider array of hydrolytic and oxidative enzymes involved in the decomposition of fungal cell wall components, such as chitin, glucans, and mannans (Janusz et al., 2017; Osono, 2007; Starke et al., 2020), and both *Metarhizium* and *Trichoderma* are fungal genera known to express these type on enzymes (Leger & Wang, 2020; Woo et al., 2023). As such, fungi may be better equipped to decompose necromass while bacterial decomposers might be more reliant on soluble fragments to rapidly access necromass resources. Previous findings (Brabcová et al., 2016, 2018; López‐Mondéjar et al., 2018) have shown bacteria utilizing resources from fungal necromass, however, these experiments were deployed in field settings or multispecies microcosm incubations in which both fungi and bacteria were present. Thus, in those experiments, fungal presence might have alleviated bacterial growth on necromass as a sole resource. Testing the aforementioned possible mechanisms, none of which are mutually exclusive, could be addressed by detailed microscopic images of decaying necromass, comparisons of decomposition dynamics using closely related fungal species that grow as either a unicellular yeast or with a hyphal morphology, as well as further enzymatic profiling.

The decoupling of necromass loss and cumulative respiration when the necromass was colonized by *Rhizobium* was also notable. Given that the soil used in the microcosms is very low in organic matter (Fernandez & Kennedy, 2018), we consider necromass to be the main source of C and nutrients in our experiment. This is supported by the higher CFUs of bacteria in necromass when compared with sand (Figure S6). The fact that bacteria did not affect mass loss over a 28‐day period, but that *Rhizobium* showed significant levels of respiration, suggests that these microbes were initially growing, but are either reliant on cell recycling or remain dormant until revived on fresh media for CFU counts. Conversely, for *Pedobacter*, which showed no mass loss nor cumulative respiration, we suspect a stoichiometric imbalance between *Pedobacter* biomass (C:N ~6) and *H. bicolor* necromass (C:N ~10; Mooshammer et al., 2014) may have contributed to its inability to grow on necromass. *Pedobacter* N content was almost double that of *H. bicolor* necromass, which might explain why this bacterium did not grow as it may have had a higher N requirement relative to the other microbial strains (Table 1). Further, of the five bacterial strains used in the Starke et al. (2020) study examining *A. bisporus* necromass decomposition, they observed *Pedobacter* growth on whole cell necromass, but not on cell wall‐enriched necromass, the latter of which is more chemically recalcitrant. Our similar findings of no *Pedobacter* growth may also be due to the greater chemical recalcitrance of *H. bicolor* necromass, which contains melanin, an aromatic molecule that has been shown to consistently slow the decomposition of fungal necromass (Beidler et al., 2020; Maillard et al., 2020).

The microbial activity metrics measured in necromass provide further insights into the mechanisms by which fungi and bacteria contribute to necromass turnover. Fungi are known to alter the pH of their local growing environment (Pawar et al., 2023) and we saw that *Metarhizium* in particular caused significant changes in necromass pH during its early colonization. Members of this genus have been found to alter local pH to facilitate the activities of enzymes involved in nutrient acquisition (Leger et al., 1998, 1999; Leger & Wang, 2020), which, in fungal necromass, would include accessing N from both cell‐soluble (e.g., proteins) and insoluble sources (e.g., chitin). The increased water content of necromass colonized by fungi relative to bacteria suggests that fungi may enhance rates of necromass decomposition by maintaining higher water availability within the substrate they are decomposing. Intriguingly, Hestrin et al. (2022) demonstrated that fungi were also critical to maintaining the activity of bacterial communities in water‐limited soils, suggesting that greater water availability in necromass may be one of the ways that fungi could enhance bacterial utilization of fungal necromass. While changes in fungal necromass chemistry during decomposition have been documented at the community level (Certano et al., 2018; Fernandez et al., 2019; Maillard et al., 2022; Ryan et al., 2020), our results link changes of specific chemical classes to specific types and strains of microbial decomposers. For example, the lower abundance of aromatic bonds in *Trichoderma*‐colonized necromass is consistent with members of this genus using oxidative enzymes to decompose organic matter substrates (Peciulyte et al., 2014). Rather than documenting direct resource utilization, we suspect the observed increase in amide bonds in both fungal colonized necromasses may be an indicator of elevated fungal decomposer abundance on necromass, similar to how increases in sterol content (likely ergosterol) were observed on decomposing fungal necromass over time in a field‐based study (Ryan et al., 2020).

While we recognize that partitioning of fungal and bacterial contributions to necromass decomposition is ecologically artificial, as both groups of microbes co‐occur on necromass throughout all stages of its decomposition, our experimental results suggest that fungal–bacterial interactions may be centrally important in facilitating bacterial degradation of necromass. Our second experiment supports this possibility, as both bacterial strains grew significantly more when co‐cultured with fungi than when grown alone in an environment with necromass as the primary C source. Bacterial growth enhancement due to the interactions with fungal partners has been attributed to active feeding on hyphal exudates or by bacterial endocellular growth within fungal hyphae (Boer et al., 2005; Leveau & Preston, 2008). For example, a *Pedobacter* strain was capable of growing exclusively in the presence of mycelial exudates (organic acids and glucose) derived from the extraradical mycelium of the arbuscular mycorrhizal fungus *Glomus* (Toljander et al., 2007). Dispersal along fungal hyphae (also known as fungal highways) (Kohlmeier et al., 2005) could also allow bacteria to overcome the exploration constraint of unicellularity and spread to new soil microsites. This could occur by bacterial attachment to actively growing mycelium or by swimming along the liquid films coating fungal hyphae. In this regard, Nazir et al. (2014) showed that a *Paraburkholderia* strain was capable of moving through hyphal networks of several fungal strains including *Trichoderma*. In addition, when co‐grown with *Paraburkholderia*, the fungus *Lyophyllum* was able to grow significantly more in the presence of antagonist bacteria and the fungicide cycloheximide. Nevertheless, in the absence of these antifungal agents, *Lyophyllum* growth with or without *Paraburkholderia* was not significantly different. This lack of bacterial effect on fungal growth is in agreement with our current findings (Figure 5). Clearly, studying fungal–bacterial interactions on decomposing necromass deserves more attention in order to unravel the mechanisms explaining the positive effect of fungi on bacterial growth.

## CONCLUSION

In this study, we demonstrated multiple types of alterations to necromass caused by fungi or bacteria growing in isolation. Collectively, our results indicate that necromass decomposition was mostly altered by microbial strain and time but not by particle size. We also showed enhanced bacterial growth in the presence of fungi, which suggests that interactions between bacteria and fungi may have an important ecological role in determining resource use from decomposing fungal necromass. Specifically, our findings indicate that fungi are significant early decomposers of fungal necromass and that when grown alone, bacteria have limited capacity to utilize necromass as a source of energy for nutrition. In the future, it will be critical to conduct experiments in which diverse members of both microbial groups are grown on necromass together and in isolation to validate the role of inter‐domain interactions. Fortunately, the dominant bacterial and fungal members of the fungal necrobiome are culturable (Kennedy & Maillard, 2023), so these kinds of experiments are readily feasible. Conducting these experiments across a range of different necromass types will also help better link the effects of varying initial substrate chemistry to necrobiome community dynamics. Ultimately, given the increasingly recognized importance of fungal necromass decomposition to soil C persistence (Angst et al., 2021; Wang et al., 2021), a mechanistic understanding of how different microbial groups individually and collectively influence rates of fungal necromass turnover will aid efforts to accurately model microbial contributions to ecosystem‐ and global‐scale C cycling.

## AUTHOR CONTRIBUTIONS

**Eduardo Pérez‐Pazos:** Conceptualization (lead); data curation (lead); formal analysis (lead); investigation (lead); methodology (lead); project administration (lead); supervision (equal); validation (equal); visualization (equal); writing – original draft (lead); writing – review and editing (lead). **Katilyn V. Beidler:** Conceptualization (lead); data curation (lead); formal analysis (lead); investigation (lead); methodology (lead); project administration (equal); validation (equal); visualization (lead); writing – original draft (lead); writing – review and editing (equal). **Achala Narayanan:** Conceptualization (lead); data curation (lead); formal analysis (lead); investigation (lead); methodology (lead); project administration (equal); supervision (equal); validation (equal); visualization (equal); writing – original draft (lead); writing – review and editing (equal). **Briana H. Beatty:** Data curation (equal); formal analysis (equal); methodology (equal); writing – review and editing (equal). **François Maillard:** Conceptualization (equal); data curation (equal); formal analysis (equal); methodology (equal); writing – review and editing (equal). **Alexandra Bancos:** Data curation (equal); methodology (equal); writing – review and editing (equal). **Katherine A. Heckman:** Data curation (equal); methodology (equal); writing – review and editing (equal). **Peter G. Kennedy:** Conceptualization (equal); data curation (equal); formal analysis (equal); funding acquisition (lead); investigation (equal); methodology (equal); project administration (equal); resources (lead); supervision (lead); validation (equal); visualization (equal); writing – original draft (lead); writing – review and editing (equal).

## CONFLICT OF INTEREST STATEMENT

The authors declare no conflicts of interest.

## Supporting information


**Fig. S1.** Glass jars microcosms. a) Lateral view of a 250 mL glass jar filled with sieved soil (*brown*) from Cedar Creek Ecosystem Science Reserve (MN, USA); the jar lid during incubations was covered with a Synthetic Filter Paper Sticker of 20 mm in diameter and 3μm mesh size, b) Top‐view of the jar showing the mycobag filled with 60 mg of gray necromass was used in each jar modifying only the grinding size, c**)** Lateral view of the jar during respiration measurements with the lid bearing a rubber septa of 20 mm in diameter. Both filter paper and rubber stopper were removed and replaced in sterile conditions.
**Fig. S2.** Necromass pH by microbial strain. Each point represents the mean of pH values by strain. Values are shown by microbial strain at 15 days post inoculation (dpi) (solid circle) and 28 (hollow circle) dpi. Error bars show ±1 standard error for that mean. Control shows the pH of uninoculated necromass.
**Fig. S3.** Necromass gravimetric water content by microbial strain. Each point represents the mean water content by strain. Values are shown by microbial strain at 15 days post inoculation (dpi) (solid circle) and 28 (hollow circle) dpi. Error bars show ±1 standard error of the mean. Control shows the water content of uninoculated necromass.
**Fig. S4.** Colony Forming Units (log_10_) by microbial strain. Each point represents the log‐transformed count of the CFU by strain. Values are shown by microbial strain at 15 days post inoculation (dpi) (solid circle) and 28 (hollow circle) dpi. Points at zero represent replicates with no CFU. Error bars show ±1 standard error.
**Fig. S5.** FTIR analyses on necromass colonized by fungi and bacteria. Panel a) FTIR spectra showing the position of each peak; b) absorbance values per peak showing peak position, the associated functional group (when available), and the chemical bond type on the faceted strip. Values are shown by microbial strain at 15 days post inoculation (dpi) (solid circle) and 28 (hollow circle) dpi for each of the eleven peaks identified. Error bars represent 95% Confidence Intervals.
**Fig. S6.** Colony Forming Units (log_10_) of *Rhizobium* when growing on necromass versus 52‐μm polyester bags filled with sand and incubated in the same soil used for the microcosm incubations in Experiment 1. Each point represents the log‐transformed count of the CFU after 14 days. Error bars show ±1 standard error.

**TABLE S1.** Three‐way Type III ANOVA on necromass loss by harvest day + particle size * strain. Asterisks indicate significance levels (n.s. non‐significant, * < 0.05, ** < 0.01, *** < 0.001).
**TABLE S2.** Two‐way Type III ANOVA on necromass loss by harvest day * strain. Asterisks indicate significance levels (n.s. non‐significant, * < 0.05, ** < 0.01, *** < 0.001).
**TABLE S3.** Analysis of Deviance Table (Type III Wald chisquare tests). Asterisks indicate significance levels (n.s. non‐significant, * < 0.05, ** < 0.01, *** < 0.001)
**TABLE S4.** Two‐way Type III ANOVA on gravimetric water content by harvest day * strain. Asterisks indicate significance levels (n.s. non‐significant, * < 0.05, ** < 0.01, *** < 0.001)
**TABLE S5.** Two‐way Type III ANOVA on gravimetric water content by harvest day * strain. Asterisks indicate significance levels (n.s. non‐significant, * < 0.05, ** < 0.01, *** < 0.001)
**TABLE S6.** Two‐way Type III ANOVA on colony forming units by harvest day * strain. Asterisks indicate significance levels (n.s. non‐significant, * < 0.05, ** < 0.01, *** < 0.001)
**TABLE S7.** One Sample t‐tests for mass loss deltas. Asterisks indicate significance levels (n.s. non‐significant, * < 0.05, ** < 0.01, *** < 0.001)
**TABLE S8.** One Sample t‐tests for gravimetric water content deltas. Asterisks indicate significance levels (n.s. non‐significant, * < 0.05, ** < 0.01, *** < 0.001)
**TABLE S9.** One Sample t‐tests for pH deltas. Asterisks indicate significance levels (n.s. non‐significant, * <0.05, **<0.01, ***<0.001)
**TABLE S10.** Two sample t‐tests for differences in FTIR absorbances between autoclaved and not autoclaved necromass. Asterisks indicate significance levels (n.s. non‐significant, * < 0.05, ** < 0.01, *** < 0.001)
**TABLE S11.** Two‐way ANOVAs for differences in FTIR absorbances by harvest day and strain. Asterisks indicate significance levels (n.s. non‐significant, * < 0.05, ** < 0.01, *** < 0.001)
**TABLE S12.** One Sample t‐tests for FTIR absorbance deltas. Asterisks indicate significance levels (n.s. non‐significant, * < 0.05, ** < 0.01, *** < 0.001)
**TABLE S13.** One Sample t‐tests for copy number deltas. Asterisks indicate significance levels (n.s. non‐significant, * < 0.05, ** < 0.01, *** < 0.001)

## Data Availability

The datasets generated during this study are available from the corresponding author on reasonable request.
