# Supplementary material for: Fungi rather than bacteria drive early mass loss from fungal necromass regardless of particle size
Source: Environ Microbiol Rep. 2024 Jun 23;16(3):e13280. doi: 10.1111/1758-2229.13280 (PMC11194057; doi:10.1111/1758-2229.13280)

**Fungi rather than bacteria drive early mass loss from fungal necromass regardless of particle size**

**Running title:** fungi drive early necromass decomposition

Eduardo Pérez-Pazos^a,b^, Katilyn V. Beidler^b^, Achala Narayanan^b^, Briana H. Beatty^b^, François Maillard^c^, Alexandra Bancos^b^, Katherine A. Heckman^d^, Peter G. Kennedy^b^

^a^Ecology, Evolution, and Behavior Graduate Program, University of Minnesota, St. Paul, MN, USA

^b^Department of Plant and Microbial Biology, University of Minnesota, St. Paul, MN, USA

^c^Microbial Ecology Group, Department of Biology, Lund University, Lund, Sweden

^d^USDA Forest Service Northern Research Station, Houghton, MI, USA

**Corresponding author:** Peter Kennedy, kennedyp@umn.edu

**Fig. S1** Glass jars microcosms. a) Lateral view of a 250 mL glass jar filled with sieved soil (*brown*) from Cedar Creek Ecosystem Science Reserve (MN, USA); the jar lid during incubations was covered with a Synthetic Filter Paper Sticker of 20 mm in diameter and 3µm mesh size, b) Top-view of the jar showing the mycobag filled with 60 mg of gray necromass was used in each jar modifying only the grinding size, c**)** Lateral view of the jar during respiration measurements with the lid bearing a rubber septa of 20 mm in diameter. Both filter paper and rubber stopper were removed and replaced in sterile conditions.


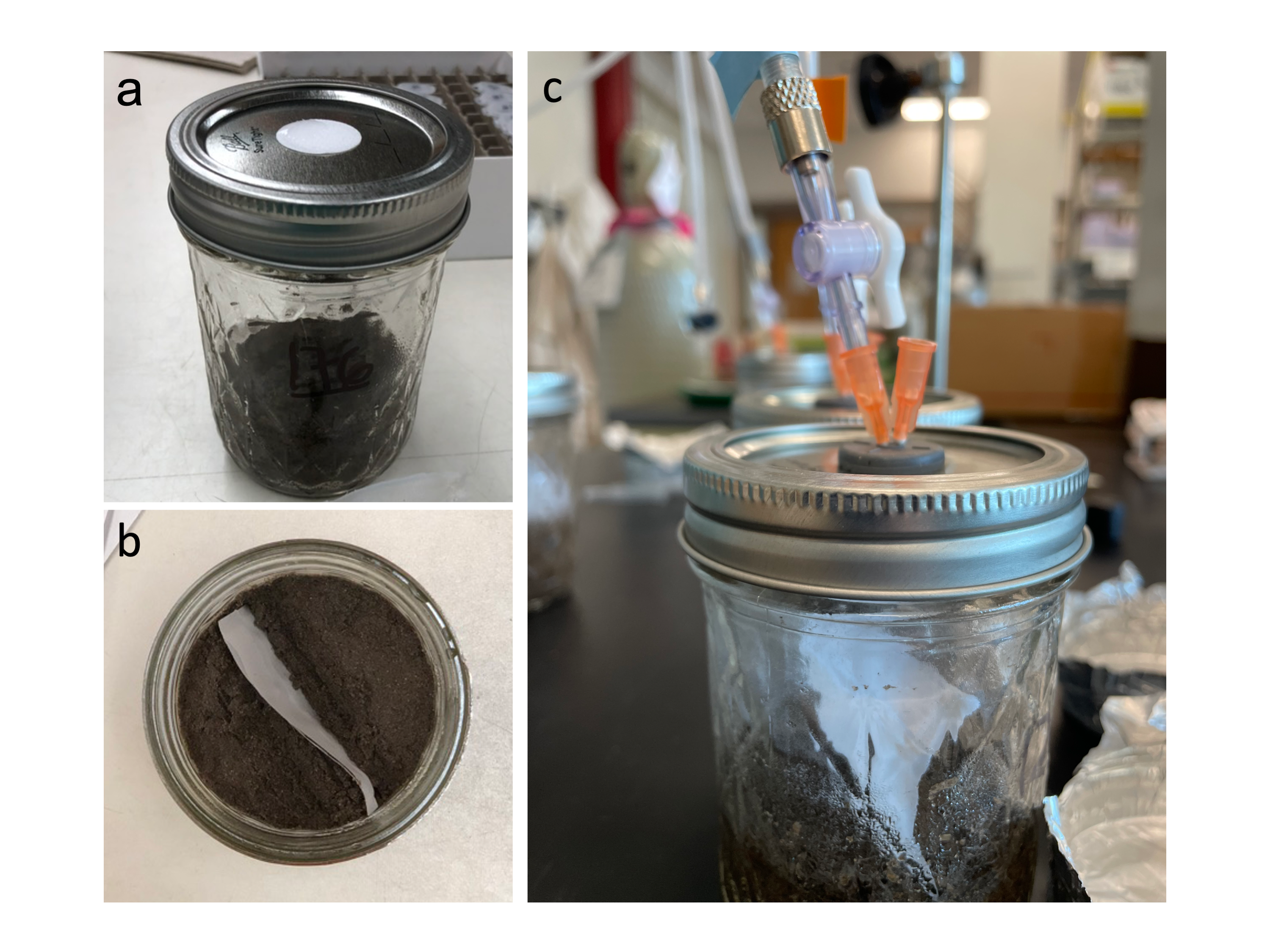


**Fig. S2** Necromass pH by microbial strain. Each point represents the mean of pH values by strain. Values are shown by microbial strain at 15 days post inoculation (dpi) (solid circle) and 28 (hollow circle) dpi. Error bars show ±1 standard error for that mean. Control shows the pH of uninoculated necromass.

**
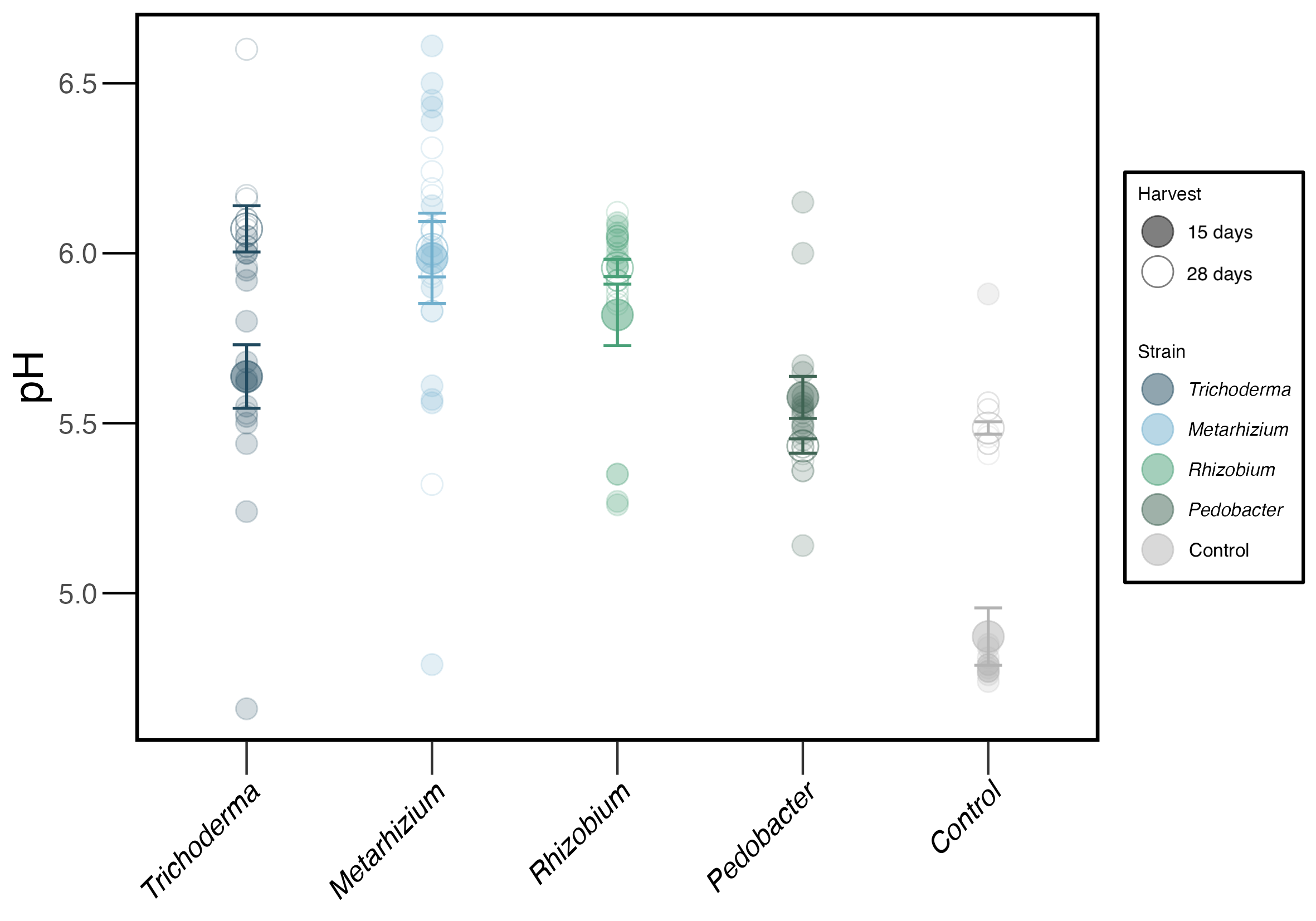
**

**Fig. S3** Necromass gravimetric water content by microbial strain. Each point represents the mean water content by strain. Values are shown by microbial strain at 15 days post inoculation (dpi) (solid circle) and 28 (hollow circle) dpi. Error bars show ±1 standard error of the mean. Control shows the water content of uninoculated necromass.

**
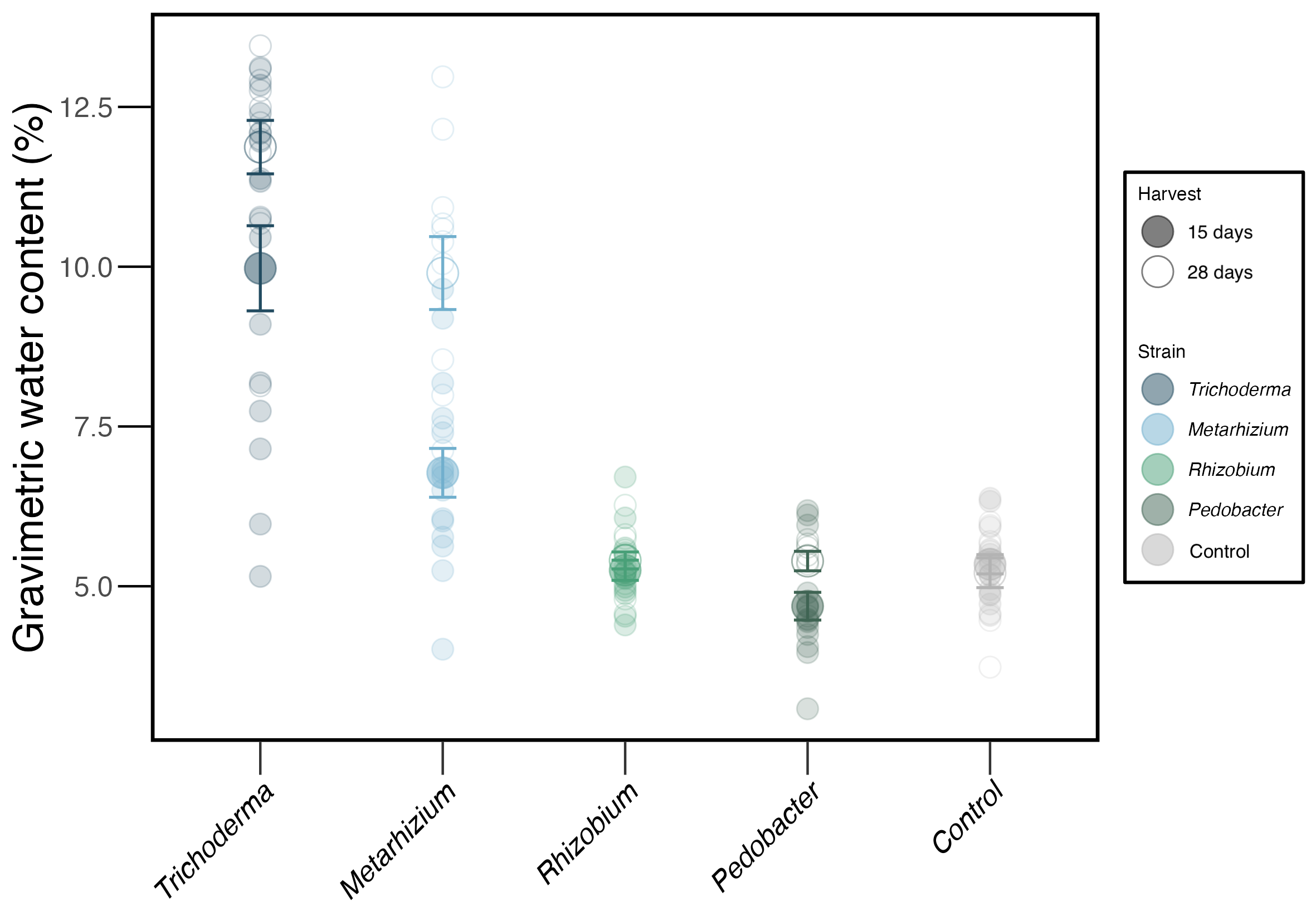
**

**Fig. S4** Colony Forming Units (log_10_) by microbial strain. Each point represents the log-transformed count of the CFU by strain. Values are shown by microbial strain at 15 days post inoculation (dpi) (solid circle) and 28 (hollow circle) dpi. Points at zero represent replicates with no CFU. Error bars show ±1 standard error.


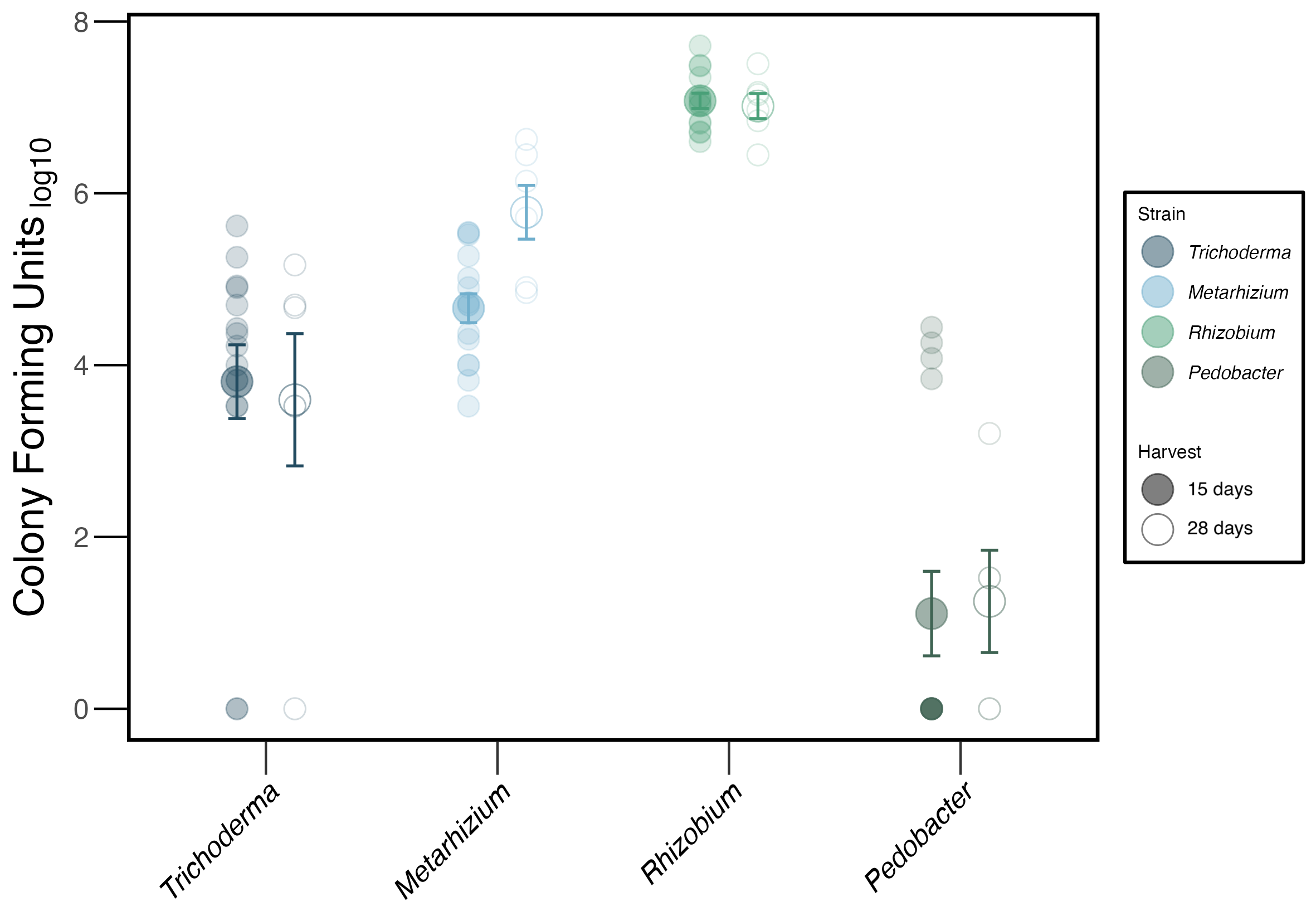


**Fig. S5** FTIR analyses on necromass colonized by fungi and bacteria. Panel a) FTIR spectra showing the position of each peak; b) absorbance values per peak showing peak position, the associated functional group (when available), and the chemical bond type on the faceted strip. Values are shown by microbial strain at 15 days post inoculation (dpi) (solid circle) and 28 (hollow circle) dpi for each of the eleven peaks identified. Error bars represent 95% Confidence Intervals.


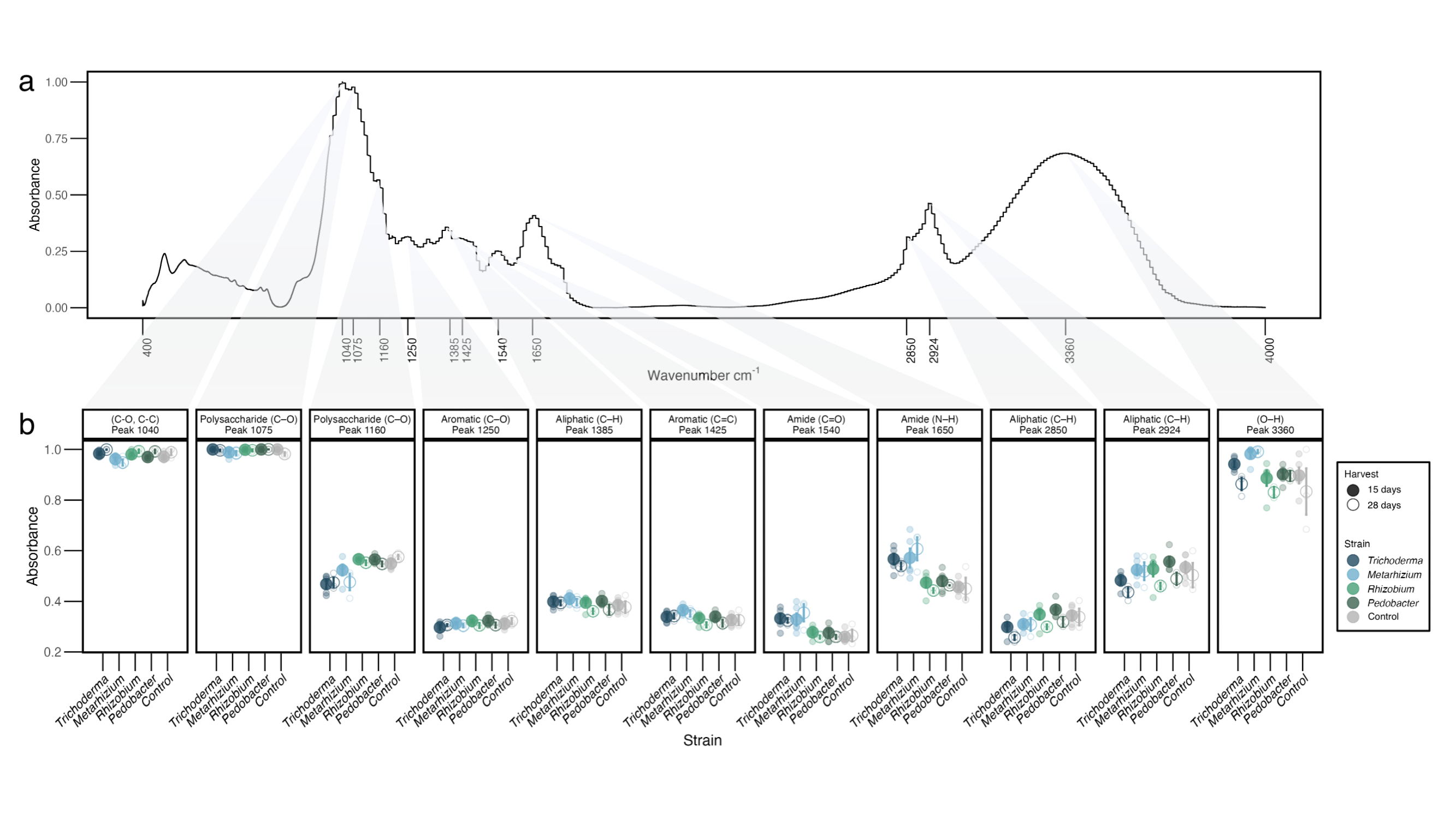


**Fig S6.** Colony Forming Units (log_10_) of *Rhizobium* when growing on necromass versus 52-µm polyester bags filled with sand and incubated in the same soil used for the microcosm incubations in Experiment 1. Each point represents the log-transformed count of the CFU after 14 days. Error bars show ±1 standard error.


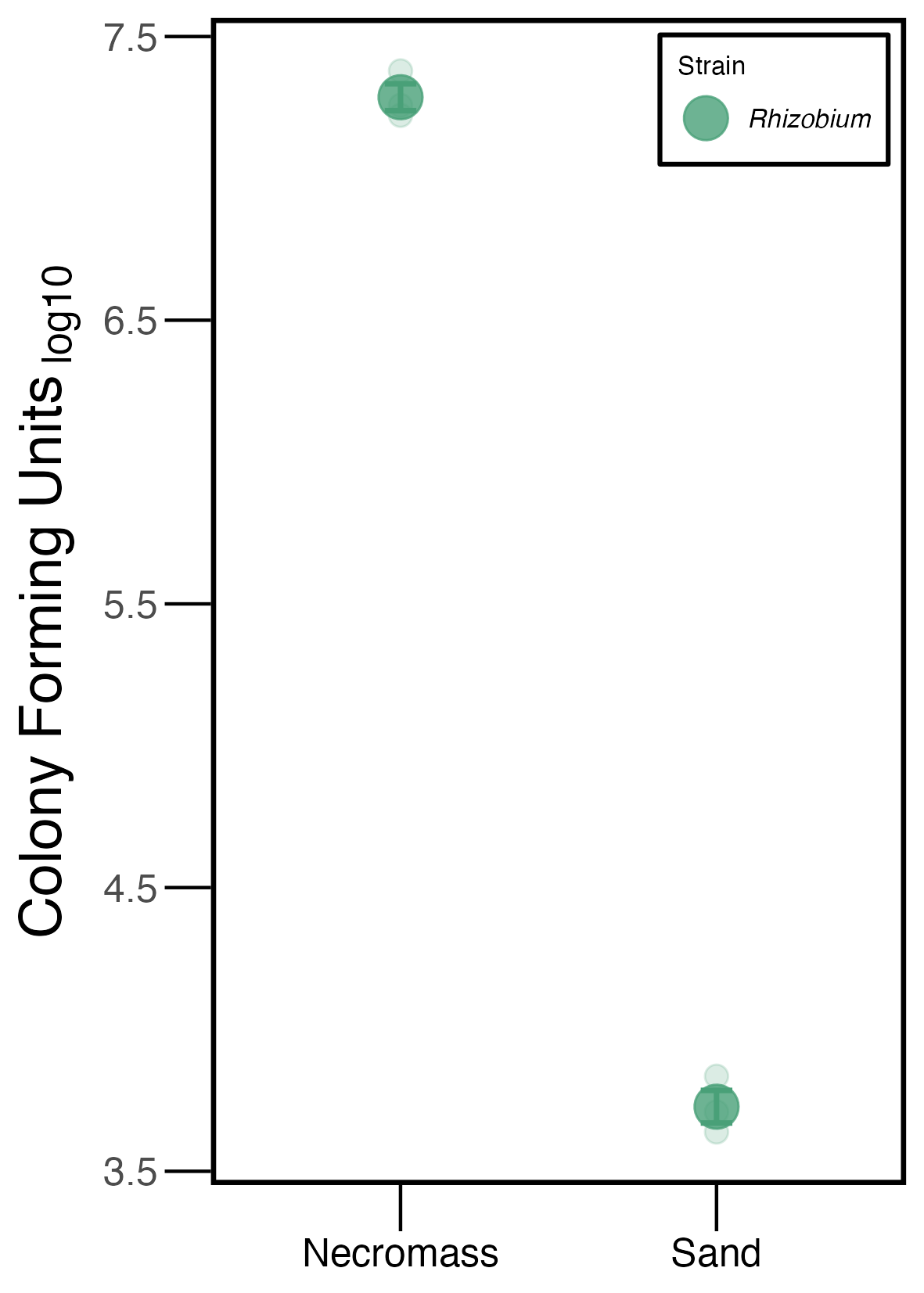

Supplement: Supplementary file 1 — Fig. S1. Glass jars microcosms. a) Lateral view of a 250 mL glass jar filled with sieved soil (brown) from Cedar Creek Ecosystem Science Reserve (MN, USA); the jar lid during incubations was covered with a Synthetic Filter Paper Sticker of 20 mm in diameter and 3μm mesh size, b) Top‐view of the jar showing the mycobag filled with 60 mg of gray necromass was used in each jar modifying only the grinding size, c) Lateral view of the jar during respiration measurements with the lid bearing a rubber septa of 20 mm in diameter. Both filter paper and rubber stopper were removed and replaced in sterile conditions. Fig. S2. Necromass pH by microbial strain. Each point represents the mean of pH values by strain. Values are shown by microbial strain at 15 days post inoculation (dpi) (solid circle) and 28 (hollow circle) dpi. Error bars show ±1 standard error for that mean. Control shows the pH of uninoculated necromass. Fig. S3. Necromass gravimetric water content by microbial strain. Each point represents the mean water content by strain. Values are shown by microbial strain at 15 days post inoculation (dpi) (solid circle) and 28 (hollow circle) dpi. Error bars show ±1 standard error of the mean. Control shows the water content of uninoculated necromass. Fig. S4. Colony Forming Units (log10) by microbial strain. Each point represents the log‐transformed count of the CFU by strain. Values are shown by microbial strain at 15 days post inoculation (dpi) (solid circle) and 28 (hollow circle) dpi. Points at zero represent replicates with no CFU. Error bars show ±1 standard error. Fig. S5. FTIR analyses on necromass colonized by fungi and bacteria. Panel a) FTIR spectra showing the position of each peak; b) absorbance values per peak showing peak position, the associated functional group (when available), and the chemical bond type on the faceted strip. Values are shown by microbial strain at 15 days post inoculation (dpi) (solid circle) and 28 (hollow circle) dpi for each of the e [file EMI4-16-e13280-s001.docx]
